# Effects of a Vibro-Tactile P300 Based Brain-Computer Interface on the Coma Recovery Scale-Revised in Patients With Disorders of Consciousness

**DOI:** 10.3389/fnins.2020.00294

**Published:** 2020-04-09

**Authors:** Nensi Murovec, Alexander Heilinger, Ren Xu, Rupert Ortner, Rossella Spataro, Vincenzo La Bella, Yangyang Miao, Jing Jin, Camille Chatelle, Steven Laureys, Brendan Z. Allison, Christoph Guger

**Affiliations:** ^1^g. tec Medical Engineering GmbH, Schiedlberg, Austria; ^2^Guger Technologies OG, Graz, Austria; ^3^g. tec Medical Engineering Spain S.L., Barcelona, Spain; ^4^IRCCS Centro Neurolesi Bonino Pulejo, Palermo, Italy; ^5^ALS Clinical Research Center, Bi.N.D., University of Palermo, Palermo, Italy; ^6^Department of Automation, East China University of Science and Technology, Shanghai, China; ^7^GIGA Consciousness, Coma Science Group, University of Liège, Liège, Belgium; ^8^French Association of Locked-in Syndrome (ALIS), Paris, France; ^9^Department of Cognitive Science, University of California, San Diego, La Jolla, CA, United States

**Keywords:** disorders of consciousness, BCI performance, tactile stimulation, P300 event-related potential, CRS-R

## Abstract

Persons diagnosed with disorders of consciousness (DOC) typically suffer from motor and cognitive disabilities. Recent research has shown that non-invasive brain-computer interface (BCI) technology could help assess these patients’ cognitive functions and command following abilities. 20 DOC patients participated in the study and performed 10 vibro-tactile P300 BCI sessions over 10 days with 8–12 runs each day. Vibrotactile tactors were placed on the each patient’s left and right wrists and one foot. Patients were instructed, via earbuds, to concentrate and silently count vibrotactile pulses on either their left or right wrist that presented a target stimulus and to ignore the others. Changes of the BCI classification accuracy were investigated over the 10 days. In addition, the Coma Recovery Scale-Revised (CRS-R) score was measured before and after the 10 vibro-tactile P300 sessions. In the first run, 10 patients had a classification accuracy above chance level (>12.5%). In the best run, every patient reached an accuracy ≥60%. The grand average accuracy in the first session for all patients was 40%. In the best session, the grand average accuracy was 88% and the median accuracy across all sessions was 21%. The CRS-R scores compared before and after 10 VT3 sessions for all 20 patients, are showing significant improvement (*p* = 0.024). Twelve of the twenty patients showed an improvement of 1 to 7 points in the CRS-R score after the VT3 BCI sessions (mean: 2.6). Six patients did not show a change of the CRS-R and two patients showed a decline in the score by 1 point. Every patient achieved at least 60% accuracy at least once, which indicates successful command following. This shows the importance of repeated measures when DOC patients are assessed. The improvement of the CRS-R score after the 10 VT3 sessions is an important issue for future experiments to test the possible therapeutic applications of vibro-tactile and related BCIs with a larger patient group.

## Introduction

**FIGURE 1:**
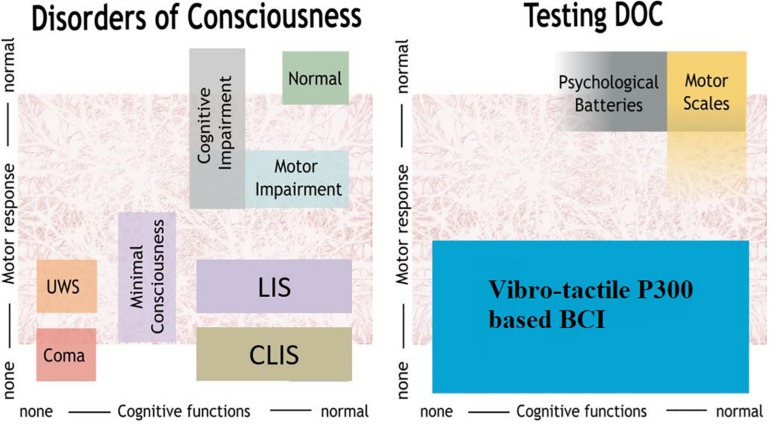
Overview of DOC and methods to assess patients. UWS, unresponsive wakefulness syndrome; LIS, locked-in syndrome, CLIS, complete locked-in syndrome; BCI, brain-computer interface.

Disorders of consciousness (DOC) include three types of patients with varying cognitive and motor functions, as shown in Figure 1. First, coma patients show closed eyes and no responsiveness to the environment. Second, patients in the unresponsive wakefulness state (UWS) present awakening (i.e., eye opening) without motor or verbal responses to command. Third, minimally conscious state (MCS) patients show inconsistent but reproducible signs of responsiveness, depending on their motor control and cognitive abilities. The fluctuation in responsiveness observed in these patients can make the diagnosis challenging. Furthermore, neurobehavioral tools used for clinical diagnosis, such as the Glasgow Coma Scale, or the Coma Recovery Scale-Revised (CRS-R) (Giacino et al., 2002; Monti et al., 2010; Risetti et al., 2013; Gibson et al., 2014), are highly dependent on voluntary motor control.

In addition to these behavioral tests, recent work has focused on brain-computer interface (BCI) systems that provide device control and communication via direct measures of brain activities (Wolpaw et al., 2002; Wolpaw and Wolpaw, 2012). EEG based on BCIs can utilize different approaches like transient evoked potentials (like the N200 or P300) (Guger et al., 2009; Lugo et al., 2014), steady-state evoked potentials (visual or somatosensory) (Bin et al., 2009; Arvaneh et al., 2016) or motor imagery (Guger et al., 2003; Blankertz et al., 2016). BCIs have been extended to help patients with DOC by assessing their mental activity and even enabling communication without movement (Lulé et al., 2013; Ortner et al., 2013, 2014; Risetti et al., 2013; Guger et al., 2017, 2018).

As many DOC patients may be unable to use interfaces based on vision, P300 BCIs using P300 BCIs using auditory and vibrotactile stimuli have been developed and proved to be on interest for assessing DOC patients (Lugo et al., 2014; Gibson et al., 2016; Erlbeck et al., 2017; Pan et al., 2018; Xie et al., 2018). However, BCI studies with DOC patients have often reported high variability and/or poor performance in a single session, and we have argued that numerous sessions are recommended to better assess a patient’s ability to use a system and to describe command following (Chatelle et al., 2018; Guger et al., 2018; Spataro et al., 2018; Allison et al., in press).

The current study explored the change in CRS-R scores before and after ten sessions of a vibrotactile BCI paradigm and changes in BCI performance across the ten sessions. Our principal aim was to explore our hypothesis that results from one or only a few sessions would not be sufficient for evaluating a patient’s cognitive capabilities and potential for BCI-based communication. Our secondary aim was to explore the possibility that training with a vibrotactile BCI paradigm could have an influence on the CRS-R score.

## Materials and Methods

### Population

This study included data acquired from 20 different DOC patients at the University of Palermo, Italy (PA) and at the Shanghai Rehabilitation Hospital 3, China (SH). The following inclusion criteria were used: patients had to be over 16 years old, and diagnosed with UWS or MCS state according to the CRS-R scale administered by experienced neurologists. Informed consent was obtained for all patients and the work was carried out in accordance with The Code of Ethics of the World Medical Association (Declaration of Helsinki).

Table 1 reports the patients’ demographic data from our convenience sample of 9 MCS patients (median age: 50, min: 26, max: 69 years) with a time since injury ranging from 4 to 48 months (median: 16), 11 UWS patients (median age: 40, min: 16, max: 67 years) with a time since injury ranging from 2 to 60 months (median: 13) and 6 healthy controls (median age: 42.5, min: 37, max: 68 years) recorded in Palermo (Spataro et al., 2018). The difference between gender and age was tested using a Chi-Square-Test (significance = *p* < 0.05). There was no difference in age and gender between the groups.

**TABLE 1:** Patient information and CRS-R scores before and after the VT3 sessions and the difference between the CRS-R scores.

**#**	**Age range (years)**	**Sex**	**Diagnosis**	**Disease duration at first session (months)**	**Mechanical ventilation**	**Clinical state**	**CRS-R before**	**CRS-R after**	**Δ CRS-R**
P1	36–40	F	CH	8	no	MCS	11	11	0
P2	21–25	F	BT	2	no	UWS	6	9	3
P3	36–40	F	TBI	13	no	UWS	5	5	0
P4	56–60	F	HBI	2	no	UWS	5	5	0
P5	26–30	F	ENC	4	yes	MCS	12	15	3
P6	16–20	F	BT	4	yes	UWS	6	7	1
P7	16–20	M	CH	11	no	UWS	6	6	0
P8	41–45	M	TBI	6	no	UWS	10	10	0
P9	16–20	M	TBI	24	no	UWS	6	8	2
P10	26–30	M	TBI	13	no	UWS	8	11	3
P11	61–65	M	IS	13	no	UWS	2	5	3
P12	56–60	M	HS	13	no	UWS	4	5	1
P13	41–45	M	HS	12	no	UWS	3	6	3
P14	66–70	M	TBI	24	no	MCS	7	9	2
P15	56–60	M	CH	20	no	MCS	7	6	−1
P16	36–40	F	A	19	no	UWS	4	4	0
P17	66–70	M	HT	60	no	UWS	4	5	1
P18	56–60	M	HT	48	no	MCS	7	9	2
P19	66–70	M	AN	16	no	MCS	13	12	−1
P20	61–65	F	SDH	48	no	MCS	7	14	7

**F, female; M, male; CH, cerebral hemorrhage; HBI, hypoxia-ischemia brain injury; SAH, subarachnoid hemorrhage; IS, ischemic stroke; HS, hemorrhagic stroke; BT, brain trauma; ENC, mitochondrial encephalopathy; SDH, subdural hematoma; HT, hematoma; AN, anoxia; UWS, unresponsive wakefulness syndrome; MCS, minimal conscious syndrome.**

### Brain-Computer Interface System

The mindBEAGLE system (Guger Technologies OG, Austria) was used to record the data and control the BCI components. Gel-based EEG electrodes were connected to a biosignal amplifier (g.USBamp, g. tec Medical Engineering GmbH, Austria). The amplifier has a 24-bit resolution with a high oversampling rate to increase the signal-to-noise ratio. The amplifier was connected to the computer via USB, and data were sent at a sampling rate of 256 Hz. The EEG signal is presented for visual inspection on a monitor during the measurement. The data are stored in floating point format for later data analysis.

EEG data were filtered with a bandpass filter between 0.1 and 30 Hz. This was done to remove baseline shifts and eliminate most EEG artifacts. The electrode positions for recording were FCz, C3, Cz, C4, CP1, CPz, CP2, and Pz according to the extended international 10–20 electrode system. The reference electrode was mounted on the right earlobe, the ground electrode was placed on the forehead. This system relies on a tactile P300 approach, where vibro-tactile tactors are placed on both wrists and foot, and subjects are instructed to silently count the stimulations based on cues provided via earbuds.

### Paradigm

A vibrotactile P300 oddball paradigm with three tactors was used (VT3). The paradigm consist of 480 stimuli per run, with 60 groups of 8 stimuli. The patient was instructed, via earbuds, to concentrate and silently count vibrotactile pulses on either the left or right wrist. A third tactor was placed on the foot to act as an additional distractor. All vibrotactile stimuli lasted 100 ms with a 100 ms pause between stimuli. The whole paradigm (i.e., one run) required around 2.5 min per run.

The patients participated in 10 sessions over 10 consecutive days. Before the 10 sessions started, each patient participated in one VT3 run to become familiar with the approach. For patients P1–P10, each session consisted of 12 VT3 runs. For patients P11–P20, each session consisted of 8 VT3 runs. There was a 1–1.5-min break between each run for all patients.

### Data Analysis

**FIGURE 2:**
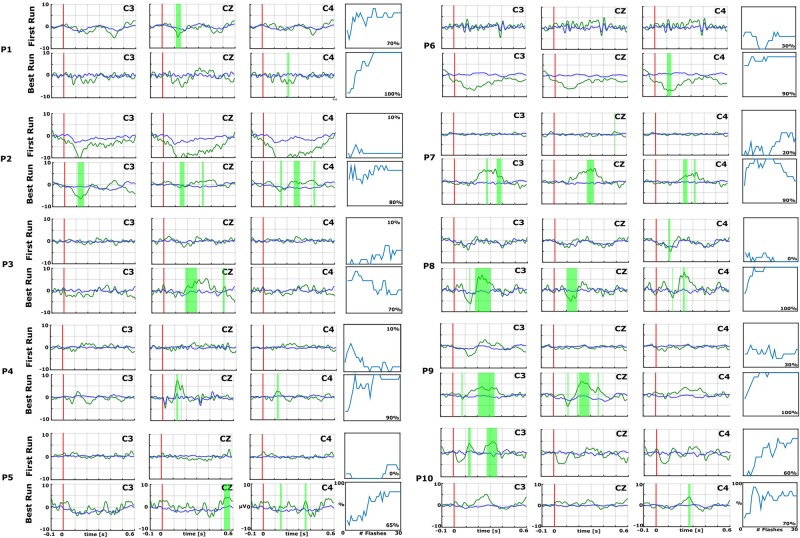
Data from the first (top) and best (bottom) runs for each patient (P1–P10) over central electrode sites (C3, Cz, and C4). In each EP plot, the red line shows stimulus onset, the blue line shows EPs from non-target trials, and the green line shows the EPs from target trials. Green shaded areas are time regions with statistically significant differences between target vs. non-target trials (*p* < 0.05). The figure to the right of each EP plot shows the classification accuracy over the number of target events. This figure presents the median accuracy for each session. The first run performed before the 10 sessions is compared with the best run during the therapy to highlight the training process.

For *post hoc* analysis, data segments of −100 ms to 600 ms around each stimulus were extracted and the EEG signal was averaged and baseline corrected. Trials with a signal amplitude ± 100 μV were rejected from further processing. The EPs were visually inspected. A linear discriminant analysis (LDA) approach was used to classify each trial. The results are presented as a classification accuracy between 0–100%, indicating how well the data could be discriminated using the classifier. In one assessment, 60 sequences of tactile stimuli were presented to the patient. Each sequence contained eight trials in randomized order. One trial within each sequence was the target trial, and the remaining seven trials were non-target trials. This resulted in 480 trials total (60 target trials/420 non-target trials, hence with a ratio of 1:7). For classification, a Fisher LDA was used without any further optimization or tuning. After removal of trials with artifacts, we then removed target and non-target trials until the 1:7 ratio was maintained again. To calculate the accuracy plots (far right side of Figure 2), the following procedure was repeated ten times, and the results were averaged into one single plot. The target and non-target trials are randomly assigned into two equal-sized pools. One pool is used to train a classifier, and the other pool is used to test the classifier. The classifier is tested on an increasing number of averaged trials out of the test pool. At first, it is tested on only one target and seven non-target trials. If the classifier detected the target stimulus correctly, the resulting accuracy was 100%, and it is 0% otherwise. The same is done for 2 averaged target trials and 14 averaged non-target trials, for 3 target trials and 21 non-target trials, and so on until the full test pool is used. This produces a plot of 30 single values (for 30 target trials in the test pool), each one either 100% or 0%. The averaging of 10 single plots results in values ranging from 0 to 100%. Increasing the number of averaged trials will increase the accuracy if the subject can follow the task, because the averaging of trials reduces random noise in the data. The accuracy value represents how well the data could be discriminated by the classifier, with a high value indicating a good separability of the EEG data.

The EPs from target and non-target trials are averaged for all channels separately. Each trial is baseline corrected before averaging, using the time segment 100 ms before stimulus onset. For each sample point, a Kruskal Wallis test (*p* < 0.05) is done to find statistical differences between target and non-target trials.

A two-way Analysis of Variance (ANOVA) was performed to test the significance of the classification accuracies. The two factors were the level of consciousness (UWS vs. MCS) and the timing of measurement (first vs. maximum accuracy). Full model ANOVA with the interaction between the two factors was first tested, followed by the focused ANOVA of each factor when significant interaction was detected. The *post hoc* comparison was performed when a main factor was detected as significant.

In addition, a one-way ANOVA was applied to the CRS-R score, and the one compared factor was the timing of measurement (pre vs. post).

## Results

### Evoked Potentials and Classification Accuracy

**FIGURE 3:**
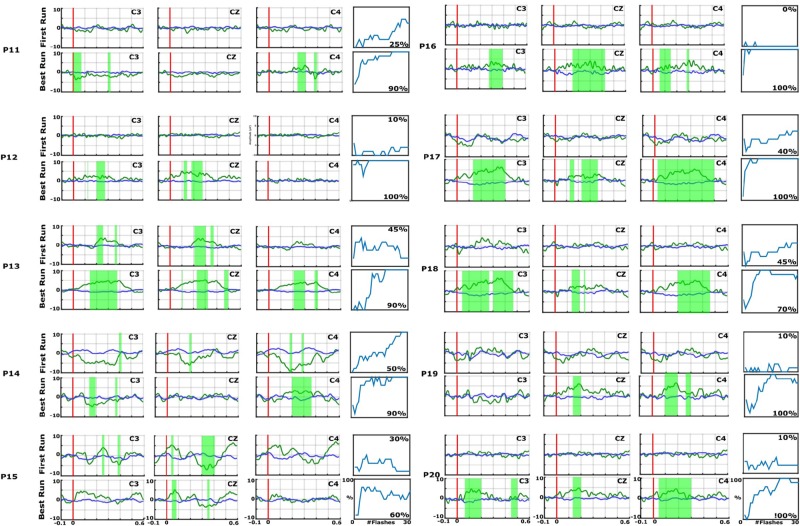
EPs and accuracies for P11–P20, with the same format as the preceding figure. In each EP plot, the red line shows stimulus onset, the blue line shows EPs from non-target trials, and the green line shows the EPs from target trials. Green shaded areas are time regions with statistically significant differences between target vs. non-target trials (*p* < 0.05). The figure to the right of each EP plot shows the classification accuracy over the number of target events. This figure presents the median accuracy for each session. The first run performed before the 10 sessions is compared with the best run during the therapy to highlight the training process.

**FIGURE 4:**
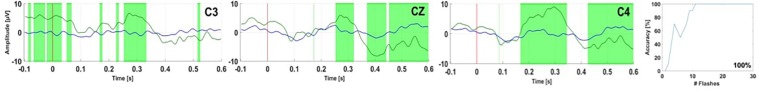
EPs and accuracy for healthy control HC 6 over electrodes C3, Cz, and C4 (Spataro et al., 2018). In each EP plot, the red line shows stimulus onset, the blue line shows EPs from non-target trials, and the green line shows the EPs from target trials. Green shaded areas are time regions with statistically significant differences between target vs. non-target trials (*p* < 0.05). The figure to the right of the EP plots shows the classification accuracy over the number of target events. This figure presents the median accuracy.

Figures 2 and 3 show the EPs and corresponding classification accuracies from all patients and Figure 4 for 1 healthy control HC 6 (Spataro et al., 2018). The EPs of the electrodes C3, CZ, and C4 are shown. Green shaded areas show significant differences between non-target and target trials. The classification accuracy is plotted from 0 to 100% over the number of vibrations of the vibro-tactile stimulator. In Figures 2 and 3 the first run performed before the 10 sessions is compared with the best run during the therapy to highlight the training process.

As an example, P7 did not show an EP in the first run, but showed a significant P300 amplitude at Cz of 5.5 μV at 340 ms after the vibration onset in the best run. C3 and C4 also show a P300. P7 achieved a median accuracy of 20% in the first run and 90% in the best run. P16 had a flat EP and an accuracy of 0% in the first run and a P300 response of 4.1 μV at 300 ms at Cz and an accuracy of 100% in the best run. C3 and C4 showed a P300 response >5.0 μV. P1 did not show a clear P300 response in the first run, but the VT3 classification accuracy achieved 70%. In the best run, only a small P300 response of 3.2 μV at 300 ms can be seen on Cz, but the classification accuracy even reached 100%.

In the first run, 10 patients had a classification accuracy above chance level (>23%). In the best run, every patient reached accuracy above chance level (minimum 60% in patient P15).

**FIGURE 5:**
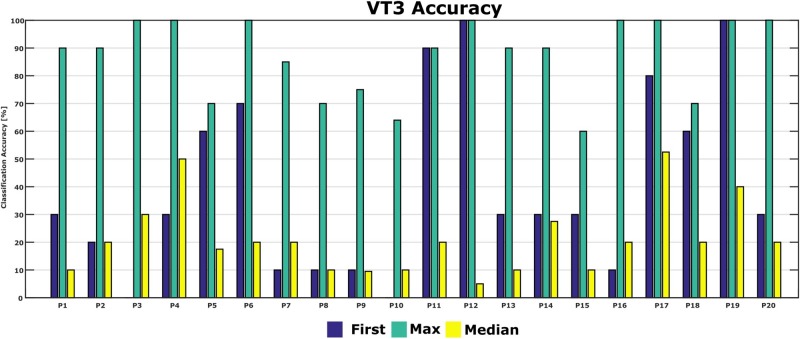
First-session, maximum and median classification accuracies for each patient. Accuracy from the first session was 0 in P3 and P10.

Figure 5 shows the classification accuracies from the first run, and the maximum and median classification accuracies over all runs across 10 days for each patient. The classification accuracy in the first run ranged from 0 to 100%. The patients achieved a maximum classification accuracy of 60% or higher. P2, P4, P6, P12, P16, P17, P19, and P20 achieved the maximum accuracy of 100% in the best run. The median accuracy ranged from 5 to 53% for all patients.

**FIGURE 6:**
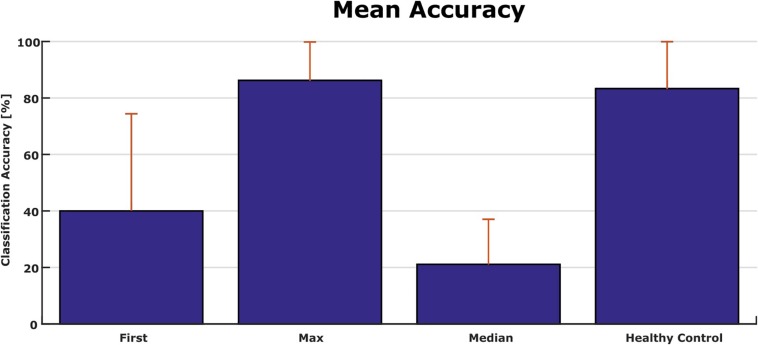
Comparison of accuracies from patients in the present study and a healthy control group with only one session [*N* = 6 (Spataro et al., 2018)].

Figure 6 shows the classification accuracy for all patients. The grand average accuracy was 40% in the first run and 88% in the best run, while the median accuracy was 21%. In comparison, a healthy control group (*N* = 6) achieved 83% classification accuracy after one VT3 run (Spataro et al., 2018).

We tested whether there was a significant difference between the classification accuracy of patients in UWS compared to patients in MCS. First, a full model ANOVA was performed and showed no interaction between the level of consciousness and timing of measurement (*p* = 0.259). Therefore, the reduced ANOVA was performed on each single classification accuracy. A significant difference was detected between classification accuracies of the first run and the classification accuracy of the best run (Mean: 40 ± 32% vs. 86 ± 14%; *p* < 0.001), while there was no statistical difference between UWS and MCS (Mean: 67 ± 36% vs. 58 ± 29%; *p* = 0.403).

### CRS-R Score

**FIGURE 7:**
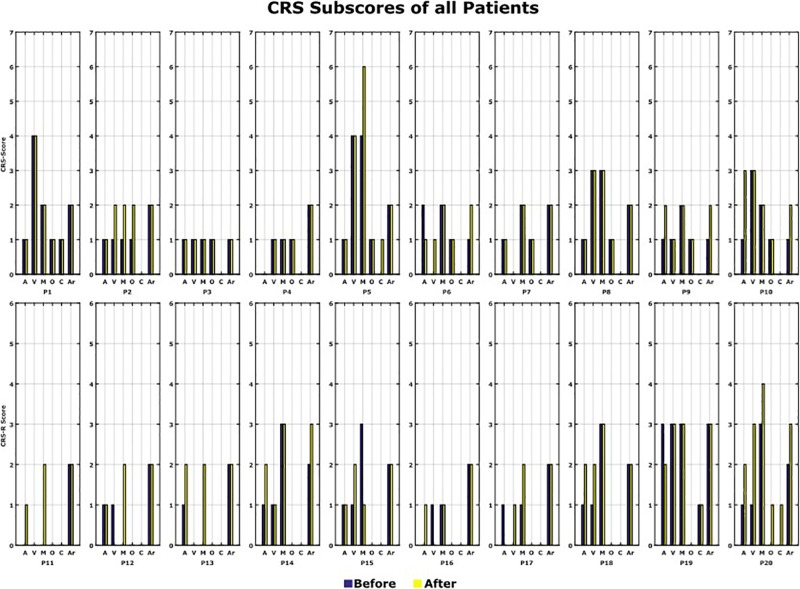
CRS-R scores. All the CRS-R subscores before and after the BCI rehabilitation therapy are shown. A, auditory function; V, visual function; M, motor function; O, oromotor/verbal function; C, communication; Ar, arousal.

The CRS-R Score was measured before and after the 10 VT3 BCI sessions. Table 1 presents the total CRS-R scores of each patient. Figure 7 shows the sub-scores of each patient.

In total, 12 out of 20 patients (P2, P5, P6, P9, P10, P11, P12, P13, P14, P17, P18, and P20) showed an improvement of one point or more after the VT3 BCI sessions (Changes in CRS-R score: Mean 2.6; Max: 7; Min: 1). Of these 12 patients, 8 improved in auditory function, 6 in visual function, 6 in motor function, 3 in oromotor/verbal function, 2 in communication and 4 in arousal.

P6, P12, and P17 had the smallest change, with a total improvement of 1 point. P20 showed the biggest improvement of 7 points. 6 patients did not show a change of the CRS-R score and 2 patients showed a decline in the score by 1 point (P15, P19).

We tested whether there is a significant CRS-R difference between patients in UWS compared to patients in MCS. First, a full ANOVA was performed, and no interaction was found between the level of consciousness and the timing of CRS-R measurement (*p* = 0.756). Therfore, the redused ANOVA was used to analyze the differences in the CRS-R scores, of the UWS and MCS patients, before the VT3 BCI sessions. A difference was found between UWS (Mean: 6.0 ± 2.0) and MCS patients as expected (Mean: 10.5 ± 2.7; *p* < 0.001), and a statistical difference was found between CRS-R score from before (Mean 6.7 ± 2.8) and after the VT3 sessions (Mean: 8.1 ± 3.1; *p* = 0.0249) for all 20 patients.

## Discussion

The aims of the study were to observe the classification accuracy across 10 VT3 sessions and to observe changes of the CRS-R score between the beginning and end of the 10 VT3 sessions.

During the 10 VT3 sessions, every patient attained a classification accuracy of 60% or higher at least once, which is well above change level. This indicates that the patients are able to follow commands using the active vibrotactile P300 paradigm used here. The results also showed that a single session is not sufficient to assess command following in DOC, since these patients have large fluctuations. This is illustrated by the relatively low classification mean accuracy of 40% in the first run, but 88% in the best run. The best run is even in the range of healthy controls if they are assessed in a single session. This highlights the importance of repeated testing of DOC patients to show command following.

An improvement in CRS-R was observed in 12 out of 20 patients, whose scores increased by 1 to 7 points. Most improvement was observed in the auditory, visual and motor subscales. The arousal and oromotor/verbal functions also improved in several patients.

The CRS-R scores may have improved for different reasons, and we do not currently claim that the tactile P300 paradigm was the principal cause. However, this possibility should be investigated. Studies have shown unexpected preservation of large-scale cerebral networks in MCS patients (McLardy et al., 1968; Hassler et al., 1969; Boly et al., 2004; Schiff et al., 2005). These findings indicate that there might be residual functional capacity in some patients that could be supported by therapeutic interventions. Prior work reported that the repetition of behavioral assessments in DOC can influence the clinical diagnosis (Wannez et al., 2017). A limitation of the study is that the CRS-R score used to control the difference was performed once before the VT3 sessions and once after the VT3 sessions. Administering the CRS-R takes a long time, which could exhaust the patient before the VT3 sessions are done. Therefore, the CRS-R was always done 1 day before or after the VT3 sessions, but not on the same day. For practical reasons, the CRS-R was not repeated several times. 18 out of 20 patients were in a stable chronic stage, and the CRS-R score was performed multiple times during their rehabilitation, whereas 2 out of 20 patients were in a subacute stage. Due to inconsistent measurements in-between the patients, these data were not presented here. Both patients in a subacute stage were UWS patients. Interestingly, one of the patients showed a 3 point improvement in the CRS-R score, whereas the other patient did not show any improvement in the CRS-R score. Disease duration did not influence the changes of the CRS-R, but of course we have only a limited sample size. The CRS-R did not change in 6 patients, and declined in 2 patients. The CRS-R declined for two MCS patients and stayed constant for one MCS patient. The biggest improvement of 7 points was achieved for an MCS patient. For 4 UWS patients, the CRS-R did not change.

The improvements in CRS-R scores may also be interpreted in the context of the “extinction of thought” perspective from prior BCI publications (Kübler and Birbaumer, 2008; Liberati and Birbaumer, 2012). Presumably, since patients who lose voluntary motor function can no longer act on their goals, they may progressively lose the ability to effect goal-directed behavior. Thus, BCIs might hypothetically counteract this “extinction of thought” by allowing patients to perform voluntary mental activities to achieve a goal. Numerous repetitions of both CRS-R assessments and an assessment/training task involving a BCI or similar approach appear to be critical for both accurate assessment and for the more tentative possibility of rehabilitation.

Results indicated that classification accuracies were significantly higher in the best run than the first run. This indicates the importance of repeated assessments with DOC patients due to the fluctuations in performance. In addition, no difference of classification accuracy was found between UWS and MCS patients, which may indicate that assessments should be repeated in both types of DOC patients.

We showed that each patient could reach ≥60% classification accuracy in at least one session. Seven patients reached a maximum classification accuracy of 100% and 6 patients reached 90%. By comparison, two studies with healthy users, which both used the same system as the current study but for only one session, reported mean VT3 accuracies of 88% (Allison et al., 2017) and 83% (Spataro et al., 2018).

The high maximum accuracies in the present study indicate that many DOC patients may be able to execute the tactile P300 paradigm in at least one session very effectively, even if performance in the first session suggests otherwise. In the first session, three of 20 patients in the present study attained accuracies of 90% or higher, three others were between 60–80%, and the remaining 14 were below 60%.

The median classification accuracy of 21% is close to other work with similar patients. Two recent studies reported a median classification of 26% (Guger et al., 2018) and 21% (Spataro et al., 2018). However, in both of these studies, only one assessment was performed with the VT3 paradigm. Although some patients reached classification accuracies >80% in these studies, we suggested that single assessments with DOC patients are not sufficient, and repeated assessments are necessary. Like similar studies with DOC patients, performance was highly variable (Chatelle et al., 2018; Guger et al., 2018; Spataro et al., 2018). This difference could be attributed to many factors in DOC patients, like motor or language impairments and vigilance fluctuations (Seel et al., 2010).

These earlier studies, as well as our present results, further support the importance of repeated measurements in DOC patients. A tactile P300-based BCI could be used as a reliable platform to repeatedly evaluate the patient’s clinical state in a short period of time. The advantages of low cost and portable setup make the EEG-based BCI system suitable for bedside measurement compared to other techniques (Cruse et al., 2012; Nicolas-Alonso and Gomez-Gil, 2012).

Single CRS-R score evaluations are not reliable in about 35% of the patients (Wannez et al., 2017), which may reflect fluctuations in conscious awareness and/or arousal that would also increase BCI performance variability. Prior work showed that cognitively mediated behavior occurs inconsistently in MCS patients, although it may be reproducible or sustained long enough to be differentiated from reflexive behavior (Giacino et al., 2002).

Prior work has explored auditory paradigms with UWS and MCS patients across BCIs that employed the MMN, P300, and/or N400 (Lulé et al., 2013; Risetti et al., 2013; Erlbeck et al., 2017). Vibro-tactile paradigms were also tested in DOC patients (Guger et al., 2018, Heilinger et al., 2018), which showed that UWS patients can control a vibro-tactile P300 BCI without prior training. 41% of all patients in this study showed signs of covert command following using the VT3 paradigm after just one assessment (Guger et al., 2018). However, these and other prior studies have usually collected data during one session.

Recent studies used transcranial direct current stimulation (tDCS) and transcranial magnetic stimulation (TMS) for treatment in UWS patients (Naro et al., 2014; Thibaut et al., 2014). TMS showed a significant clinical improvement in some patients, while tDCS treatment presented no significant results. Although TMS entails some serious health risks in inducing seizures (Rossi et al., 2009), a BCI or similar approach could complement TMS or provide an alternative approach for non-pharmacological treatment of these patients. Future studies are needed to explore if TMS or tDCS in combination with the tactile BCI paradigm presented here could affect the possible training outcomes.

More broadly, tactile P300 paradigms and related approaches could be a complement and/or alternative to many emerging treatment approaches that have been explored to facilitate emergence into consciousness, such as TMS, TDCS, thalamic stimulation, pharmacological intervention, or spinal cord stimulation (Ragazzoni et al., 2017; Schnakers and Monti, 2017; Kundu et al., 2018; Thibault et al., 2018). However, none of the therapeutic options currently available are highly effective (Giacino et al., 2018), and thus there is a strong need to evaluate new options with patients. Physical therapy can impact spasticity and muscle contracture in DOC patients (Thibault et al., 2018), which could comprise an additional component of an overall treatment approach.

This study is limited by the small sample size of 20 patients. Future work will explore the CRS-R score and BCI accuracy across multiple sessions with more patients and controls. Another limitation that we noted is the assessment of the CRS-R only twice (before and after the 10 recording sessions). In future work, the CRS-R test may be performed repeatedly within the 10 sessions to better assess fluctuations in CRS-R score.

## Summary

Results suggest that several sessions with the vibrotactile P300 paradigm and CRS-R are necessary, due to the high variability within this patient group. Several patients who performed poorly in the first session could instead execute tasks, and potentially communicate with a BCI, based on results from later sessions. Future work should explore the speculative suggestion that BCI training might potentially have a therapeutic impact on DOC patients. This would require a larger effort than the current study, with matched controls.

## Data Availability Statement

The datasets generated for this study are available on request from the corresponding author.

## Ethics Statement

The studies involving human participants were reviewed and approved by the University of Palermo, Italy and Shanghai Rehabilitation Hospital 3, China. Written informed consent to participate in this study was provided by the participants’ legal guardian/next of kin.

## Author Contributions

NM, AH, and YM were mainly responsible for data collection which occurred in collaboration with RS and JJ. NM and AH were mainly responsible for data analysis and manusript preparation. The remaining authors were responsible for study design and manuscript preparation.

## Conflict of Interest

NM and CG are employed by Guger Technologies OG, which is the company that built the mindBEAGLE system. NM, AH, RX, CG, and RS are employed by g. tec Medical Engineering GmbH. RO is employed by g. tec Medical Engineering Spain S.L. SL is on the scientific advisory board of g.tec. The remaining authors declare that the research was conducted in the absence of any commercial or financial relationships that could be construed as a potential conflict of interest.
